# Hemoglobin level trajectories in the early treatment period are related with survival outcomes in patients with breast cancer

**DOI:** 10.18632/oncotarget.13679

**Published:** 2016-11-29

**Authors:** Chia-Lin Lee, Chun-Hao Tsai, Dah-Cherng Yeh, Chi-Shy Lin, Yu-Fen Li, Huey-En Tzeng

**Affiliations:** ^1^ Division of Endocrinology and Metabolism, Department of Internal Medicine, Taichung Veterans General Hospital, Taiwan; ^2^ Department of Public Health, College of Public Health, China Medical University, Taichung, Taiwan; ^3^ Department of Medical Research, Taichung Veterans General Hospital, Taiwan; ^4^ Graduate Institute of Biostatistics, China Medical University, Taichung, Taiwan; ^5^ Department of Orthopedics, China Medical University Hospital, Taichung, Taiwan; ^6^ School of Medicine, China Medical University, Taichung, Taiwan; ^7^ Department of Surgery, Taichung Tzu Chi General Hospital, Taichung, Taiwan; ^8^ Department of Family Medicine, Taichung Veterans General Hospital, Taiwan; ^9^ Division of Hematology/Oncology, Department of Medicine, Taichung Veterans General Hospital, Taichung, Taiwan; ^10^ Breast Cancer Center, Taichung Veterans General Hospital, Taiwan

**Keywords:** breast cancer, hemoglobin, survival, trajectory

## Abstract

Hemoglobin (Hb) levels are reportedly related with treatment outcomes and survival in patients of breast cancer. However, the long-term change in Hb levels after treatment and the effects of Hb on survival remain unknown. This retrospective cohort study enrolled 1931 breast cancer patients with pathological stage I-IV between 1/1/2003 and 12/31/2013. Latent class modeling was used to identify trajectories in monthly Hb levels over time. The primary endpoint was 10-year cancer-related death. We identified 5 distinct Hb trajectories: persistent anemia (5.6 %; n = 109), improved anemia (4.8 %, n = 93), mild anemia (21.0%; n = 406), low normal Hb (46.6 %; n = 899), and normal Hb (21.9%; n = 424). Compared with the normal-Hb group, trajectories with low Hb levels had worst 10-year survival. The adjusted hazard ratios were 1.79(95% CI, 0.91-3.53) for the improved anemia group, 1.09(95% CI, 0.68-1.74) for the mild anemia group, 1.06 (95% CI, 0.71-1.60) for the low normal Hb group, and 2.19(95% CI 1.28-3.75) for the persistent anemia group. Our findings show there are five Hb level trajectories during breast cancer treatment. The anemia Hb level trajectory during the first 12 months after treatment reflect the worst cancer-related 10-year survival in breast cancer patients.

## INTRODUCTION

The incidence of anemia in patients with solid tumors is reportedly 2–78% [1]. The causes of anemia in cancer patients are multifactorial, including chemotherapy and radiation-induced myelosuppression, bleeding, marrow infiltration by cancer invasion, nutritional deficiencies, and cytokine-mediated anemia. Hemoglobin (Hb) levels are related with treatment outcomes and survival in patients with various cancers [2–8]. However, there are few studies that have focused on pre-treatment Hb levels for breast cancer prognosis [9–13]. Moreover, the Hb level might change after treatment (chemotherapy, radiation therapy, or surgery) or with different cancer stages. Therefore, the long-term effects of Hb on survival after treatment remain unknown.

We hypothesized that multiple Hb level trajectories exist within different stages of breast cancer, and a lower Hb level trajectory is associated with poorer long-term survival.

Therefore, the aims of this study were to identify subgroups with similar Hb level trajectories with different breast cancer stages, to determine the independent association cancer stages level trajectories within a specific treatment time interval and long-term survival, and to assess survival with different cancer stages based on Hb trajectories.

## RESULTS

### Characteristics of patients

Table 1 summarizes the basic characteristics of the study population. Of the 1931 patients who were enrolled, 256 patients died during the follow up. The mean age of the patients at the time of diagnosis was 52 years; most of the patients were aged 40–49 years (33%). Primary breast cancer diagnoses occurred at stage II for 43.5% of the patients, at stage I for 31.0% of the patients, and at stage III for 22.3% of the patients. Surgery was the most common treatment for patients following diagnosis (99.9%), followed by chemotherapy (70.2%) and radiotherapy (55.1%). During the 5 years following the primary treatment, 64.4% of the cases received hormone therapy.

**Table 1:** Baseline characteristics

Variable	Mean±SD or N(%)
Age at diagnosis	
<40	249(12.89)
40-49	635(32.88)
50-59	598(30.97)
Over60	449(23.25)
Pathologic stage	
1	596(30.86)
2	840(43.5)
3	430(22.27)
4	65(3.37)
ER+	1249(65.09)
PR+	1000(52.14)
ER+ or PR+	1334(69.52)
HER2	614(35.66)
Cell Differentiation	
well	200(10.36)
moderate	951(49.25)
poor	780(40.39)
Hb at diagnosis	12.8±1.4
Surgery	1928(99.9)
Chemotherapy	1351(70.0)
Radiotherapy	1064(55.13)
Parity Hx	1640(88.60)

Abbreviations: ER+, estrogen receptor positive. PR+, progesterone receptor positive. ER+ or PR+, estrogen or progesterone receptor positive. HER2, human epidermal growth factor receptor 2 positive.Hb, hemoglobin. Hx, history.

### Trend in Hb changes within one year of treatment

**Figure 1:**
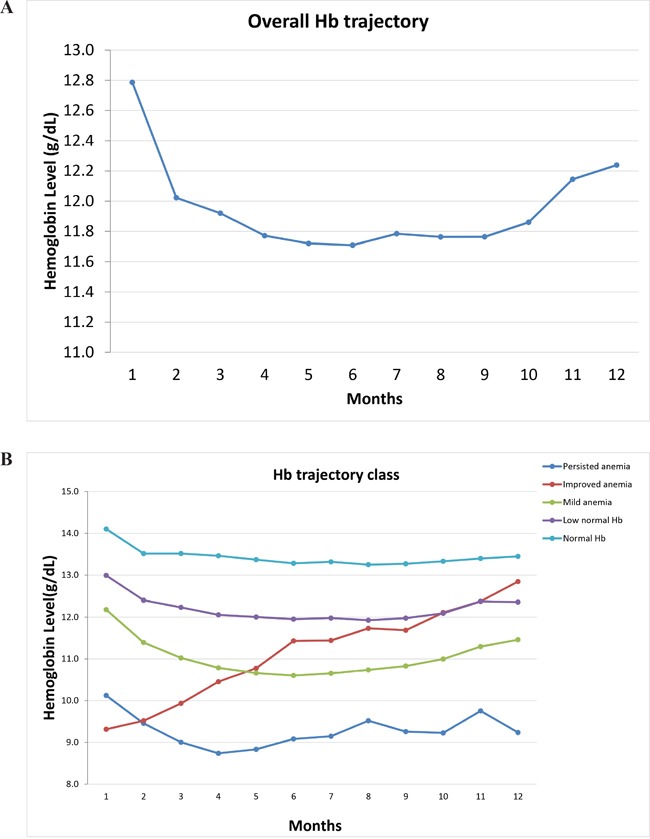
Trajectory of Hb level within the first year of breast cancer Overall Hb trajectory (1**A**) and distinct five Hb trajectories (1**B**) by group-based trajectory modeling were plotted.

The mean Hb level was the highest at the beginning of the 1-year period following treatment and then gradually decreased to <12.0 mg/dL at 3 months and returned to >12.0 mg/dL at the end of the 1-year period (Figure 1A).

### Changes in different Hb trajectories

Using the group-based trajectory modeling, we identified five Hb trajectories within the 1-year period following treatment (Figure 1B, Table 2, Supplementary Figure S1). There were no differences in cell differentiation grade and parietal history between the trajectories. The normal Hb group had the highest baseline Hb level and a higher percentage of pathological stages I and II and was more likely to have a ER/PR(+) status and to have received hormone therapy. A higher proportion of the persistent anemia group had stage III or IV cancer, and the Hb level was slightly higher in the persistent anemia group than in the improved anemia group.

**Table 2:** Baseline Characteristics stratified by Hb trajectory

Variable	Persisted anemia (N=109)	Improved anemia (N=93)	Mild anemia (N=406)	Low normal Hb (N=899)	Normal Hb (N=424)	P value
Pathologic Stage						<0.001
1	18.4	23.7	20.7	35.3	36.1	
2	40.4	44.1	45.8	40.9	47.4	
3	33.0	25.8	29.3	20.8	15.1	
4	8.2	6.4	4.2	3.0	1.4	
ER+	63.9	68.5	62.7	63.9	69.5	0.22
PR+	53.7	58.2	47.9	51.6	55.6	0.17
ER+ or PR+	71.3	75.0	65.9	68.7	73.1	0.15
HER2	37.2	32.2	41.0	34.6	33.2	0.16
Hormone therapy	67.0	67.7	61.6	63.3	67.9	0.29
Chemotherapy	71.6	81.7	82.3	66.4	63.0	<0.001
Surgery	100.0	97.9	100.0	100.0	100.0	<0.001
Parity Hx	95.2	88.6	86.7	88.3	89.5	0.18
Age at diagnosis						<0.001
<40	11.0	8.6	13.3	14.4	10.6	
40-49	34.9	64.5	32.0	31.7	28.8	
50-59	22.0	21.5	30.8	30.5	36.5	
Over60	32.1	5.4	23.9	23.4	24.1	
Radiation therapy	54.1	60.2	64.0	54.0	48.1	<0.001
Cell Differentiation						0.30
Well	10.1	10.7	8.4	10.6	11.8	
Moderate	43.1	53.8	46.5	50.0	50.7	
Poor	46.8	35.5	45.1	39.4	37.5	
Hb at diagnosis	10.4±1.3	9.6±1.4	12.3±0.9	13.1±0.7	14.2±0.6	<0.001

Data are presented with percentage for categorical data and Mean±SD for continue variable.

Abbreviations: ER+, estrogen receptor positive. PR+, progesterone receptor positive. ER+ or PR+, estrogen or progesterone receptor positive. HER2, human epidermal growth factor receptor 2 positive. Hb, hemoglobin.

### Cancer-related survival based on Hb trajectory

**Figure 2:**
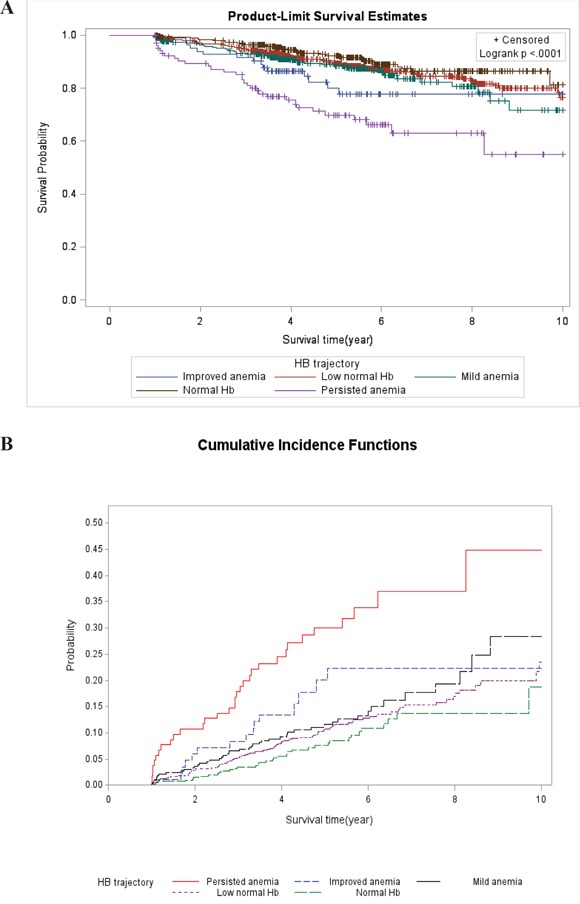
The 10-year survival curve according to the trajectory groups The highest mortality rate occurred for the persistent anemia group. The highest survival rate occurred for the normal Hb group. Survival curve of Kaplan-Meier analysis by Hb trajectory is shown (2**A**). The cumulative incidence by trajectory groups is plotted (2**B**).

The 10-year cancer-related survival curve according to the trajectory groups is shown in Figure 2A. The cumulative incidence by trajectory groups is plotted in Figure 2B. The highest mortality rate occurred for the persistent anemia group. The highest survival rate occurred for the normal Hb group. Compared with the normal Hb group, the persisted anemia group had significantly increased mortality in the univariable and multivariable analysis. Nevertheless, the improved anemia group increased mortality in the univariable, but not in the multivariable cox proportional hazards analysis (Table 3). In general, the higher Hb group had a trend for improved mortality (p for trend <0.001). Although there were no differences in differentiation grade among the Hb trajectories (Table 2), poor cell differentiation grade was associated with a poor 10-year survival rate. Regarding the impact of the Hb trajectories on survival, in the univariable and multivariable analysis, mortality began to significantly increase after 1 year for the persisted anemia group (Table 4). In the multivariable analyses, mortality did not significantly increase for the mild anemia, improved anemia and low normal Hb groups.

**Table 3:** Survival analysis of factors associated with overall mortality by cox-proportional hazard model

Variable	Univariate		Multivariate		P for trend
	HR (95% C.I)	P value	HR (95% C.I)	P value	
Hb trajectory					<0.001
Normal Hb	Ref.	-	Ref.	-	
Persisted anemia	4.17(2.59-6.71)	<0.001	2.19(1.28-3.75)	<0.01	
Improved anemia	2.19(1.20-4.00)	0.01	1.79(0.91-3.53)	0.09	
Mild anemia	1.59(1.03-2.45)	0.04	1.09(0.68-1.74)	0.73	
Low normal Hb	1.34(0.92-1.96)	0.13	1.06(0.71-1.60)	0.77	
HER2	1.25(0.95-1.66)	0.12	0.65(0.48-0.90)	<0.01	
ER or PR (+)	0.53(0.41-0.69)	<0.001	0.44(0.31-0.61)	<0.001	
Parity Hx	0.53(0.38-0.76)	<0.001	0.56(0.37-0.84)	<0.01	
Pathologic Stage					<0.001
1	Ref.	-	Ref.	-	
2	2.10(1.29-3.43)	<0.01	1.95(1.06-3.59)	0.03	
3	6.75(4.22-10.79)	<0.001	6.68(3.58-12.45)	<0.001	
4	32.59(19.05-55.76)	<0.001	40.41(20.41-80.02)	<0.001	
Age at diagnosis					0.11
<40	Ref.	-	Ref.	-	
40-49	0.65(0.43-1.00)	0.05	0.74(0.46-1.19)	0.21	
50-59	1.06(0.71-1.60)	0.77	1.07(0.67-1.71)	0.77	
Over60	1.30(0.86-1.96)	0.22	1.34(0.82-2.19)	0.25	
Cell Differentiation					0.24
Well	Ref.	-	Ref.	-	
Moderate	5.82(2.14-15.79)	<0.001	3.18(1.16-8.71)	0.02	
Poor	7.81(2.89-21.13)	<0.001	2.96(1.07-8.19)	0.04	
Surgery	0.16(0.02-1.12)	0.06	1.39(0.17-11.53)	0.76	
Chemotherapy	2.09(1.50-2.92)	<0.001	0.82(0.51-1.31)	0.41	

Abbreviations: ER+, estrogen receptor positive. PR+, progesterone receptor positive. ER+ or PR+, estrogen or progesterone receptor positive. HER2, human epidermal growth factor receptor 2 positive. Hb, hemoglobin.

**Table 4:** Hazard ratios of the association of Hb trajectory groups with mortality at different time points

Variable	2 year		3 year		5 year		10 year	
	Univariate	Multivariate	Univariate	Multivariate	Univariate	Multivariate	Univariate	Multivariate
	HR (95% C.I)	HR (95% C.I)	HR (95% C.I)	HR (95% C.I)	HR (95% C.I)	HR (95% C.I)	HR (95% C.I)	HR (95% C.I)
Hb trajectory								
Normal Hb	Ref.	Ref.	Ref.	Ref.	Ref.	Ref.	Ref.	Ref.
Persisted anemia	7.8(2.9-21.1)**	3.4(1.2-10.2)*	5.6(2.8-11.3)**	2.9(1.3-6.2)*	4.9(2.9-8.3)**	2.4(1.3-4.3)*	4.2(2.6-6.7)**	2.2(1.3-3.8)*
Improved anemia	4.0(1.2-13.2)*	2.1(0.5-8.8)	2.5(1.0-6.1)	1.6(0.5-4.5)	2.7(1.4-5.3)*	2.0(0.9-4.2)	2.2(1.2-4.0)*	1.8(0.9-3.5)
Mild anemia	2.5(0.9-6.6)	1.5(0.6-4.1)	1.9(1.0-3.8)*	1.2(0.60-2.5)	1.7(0.9-2.7)	1.0(0.6-1.8)	1.6(1.0-2.5)*	1.1(0.7-1.7)
Low normal Hb	1.9(0.8-4.8)	1.3(0.5-3.4)	1.5(0.8-2.8)	1.1(0.6-2.1)	1.5(0.9-2.3)	1.0(0.6-1.6)	1.3(0.9-2.0)	1.1(0.7-1.6)

* P value<0.05.

** P value<0.001.

Multivariate: adjusted for age at diagnosis, pathologic stage, cell Differentiation, surgery, chemotherapy, parietal history, estrogen or progesterone receptor positive, human epidermal growth factor receptor 2 positive.

## DISCUSSION

The current study revealed there are 5 unique trajectories of Hb levels during the first year after treatment. The persisted anemia group (initial Hb level of approximately 10 g/dL) were significantly associated with worst 10-year survival compared with the normal Hb trajectory groups (Hb threshold of approximately 12-14 g/dL) in breast cancer patients.

It is notable that most Hb levels declined in the first 6 months after treatment initiation, but the Hb level continued to increase after treatment in the improved anemia trajectory. Despite slightly lower initial Hb levels in the improved anemia group than in the persistent anemia group, the improved anemia group had better long-term survival, with improved survival after 3 years; meanwhile, the mild anemia, low normal Hb, and normal Hb groups had significantly improved survival after 2 years.

Hypoxia-inducible factor-1 (HIF-1), which is a key molecular response to hypoxia, leads to structural and functional abnormalities in the tumor microvasculature and exacerbates the progress through the pathologic stages [14–17]. HIF-1 contributes to the cancer biology including angiogenesis [18–20], epithelial-mesenchymal transition [21–23], invasion [24, 25], metastasis [26–28], resistance to radiation therapy and chemotherapy [29–31]. Therefore, a recent study showed that HIF-1α is an indicator of tumor progression, metastasis, and poor patient prognosis. Higher expression of HIF-1α is correlated with poorer survival in breast cancer patients [32, 33]. In contrast, the benefit of higher Hb levels during treatment might be the result of several factors such as increased blood flow and drug delivery to the tumor or the higher effectiveness of radical-generating agents in the presence of a better oxygen supply, as postulated for radiotherapy [34].

There are several factors that reflect the treatment outcome or survival in breast cancer, including cell differentiation grade, parietal history, ER/PR status, and pathological stage.

Hemoglobin (Hb) levels are related with treatment outcomes and survival in patients with various cancers. However, the anemia criteria of Hb level differs from literatures. A meta-analysis study by Caro JJ et al. showed anemia is associated with poor survival in cases with lung carcinoma, cervicouterine carcinoma, head and neck carcinoma, prostate carcinoma, lymphoma, and multiple myeloma. However, the cut-off point criteria of anemia varied from 8.5 g/dL to 14.0 g/dL [2]. Moreover, the Hb level might change after treatment (chemotherapy, radiation therapy, or surgery) or with different cancer stages. The timing of asses Hb level associated outcome also vary, including preoperatively, the lowest point during treatment and various time point after therapy. For example, preoperative anemia has proved associated with poor prognosis in breast cancer [13]. The pre-treatment Hb level (cut-off 12 g/dL) was an independent prognostic factor for overall survival in anal canal cancer patients after radiation-chemotherapy [35]. Pretherapeutic Hb level as an independent useful marker for predicting pathologic tumor response in esophageal squamous cell carcinoma [4].

However, Hb at the beginning of radiotherapy for supraglottic larynx cancer does not correlate with treatment outcome, but decrease of Hb during therapy is a strong prognostic factor for treatment failure [3]. Ye et al. confirmed the prognostic importance of hemoglobin level during chemotherapy in gastric cancer patients [5]. Hb values (cutoff 12.45 g/dL) at the last week of radiation therapy was an independent prognostic factor for overall survival, failure-free survival and loco-regional failure-free survival in nasopharyngeal carcinoma patients [36]. The information from literature was limited to analyze the relation between different hemoglobin levels change pattern and survival. The Hb trajectory can also explain why increasing the Hb level by transfusion or erythropoietin stimulation did not result in improved outcome for patients with low initial hemoglobin levels in head and neck squamous cell carcinoma [8]. The trend of Hb trajectory over time can reflect pre-treatment health status and post-treatment response and could be classified into different groups. Therefore, we need a comprehensive view of overall course in Hb change for future intervention. This study with its longitudinal design and a homogenous population made it possible to investigate patterns of behavioral adjustment since the treatment of breast cancer.

Our data revealed that Hb level is a reliable indicator for response to therapy; the initial Hb level after treatment provided an accurate survival prediction, and the change in Hb level was associated with the response to treatment. Although the initial Hb level in the improved anemia group was approximately 9g/dL, an increasing trend in Hb level reflected good response to treatment and resulted in better 10-year survival, compared with the persistent anemia group.

Latent class modeling [37, 38], as used in the current analyses, provides unique insight into Hb level trajectories during the first 1 year post-treatment period as they relate with 10-year overall survival. The current study confirmed the prognostic importance of different Hb trajectories as well as a more realistic understanding of the separate trajectory groups. We extended those findings to demonstrate not only that prognosis differs by Hb level but also that these trajectories reflect survival. Therefore, the trend in the change in Hb level is more important than the level at a single time during the treatment of breast cancer. This understanding of the effect of a change or timing of the change in Hb level in various cancer stages might be important for the stratification of survival predictions and future intervention decisions.

Whether preoperative anemia and chemotherapy-induced anemia are both associated with poor prognosis of patients with breast cancer remains to be clarified. In addition, controversy remains regarding adequate Hb levels and the benefits of red cell transfusion, erythropoietic stimulating agents, or iron supplement therapy in the treatment of breast cancer-related anemia [5, 39–42]. The current results provide a long-term perspective of different Hb groups for therapy intervention.

The strength of our study is the use of a trajectory method which has performed in cardiovascular and cerebrovascular disease [43, 44] To our knowledge, this is the first paper about Hb trajectory related with solid cancer survival. There is also a large number of patients with follow up for twelve years. However, several limitations must be considered when interpreting these findings. Our data only included overall survival but did not provide other prognosis information such as local relapse-free survival, lymph node metastasis-free distance metastasis free survival, and relapse-free survival. Therefore, we were unable to determine whether Hb level trajectories play the same role in the prediction of these other types of survival. Second, we used a 1-year Hb level trajectory rather than another time interval because these patients would not have been followed up monthly 1 year after treatment; instead, most of the patients were followed up every 6 months starting1 year after treatment. Third, we were not able to assess the effect of blood transfusion while treatment; we lacked information regarding the need and criteria for transfusion because they differ by patient condition and physician decision. The question of the best timing to increase Hb levels or the effect for patients with preoperative anemia remains unanswered. Therefore, further studies are needed.

Our findings revealed that different Hb level trajectories in the first year after treatment predict long-term cancer-related survival. This analysis revealed that improving Hb levels reelected better cancer-related survival in breast cancer patients. Additional research is needed to examine the utility of specific Hb trajectories in clinical events, the treatment, and timing of anemia intervention for breast cancer patients.

## MATERIALS AND METHODS

### Data source

This retrospective cohort study enrolled breast cancer patients from the Taichung Veterans General Hospital, a medical center in central Taiwan. All female cases of pathologically proven breast cancer during 2003–2011 were included.

Age at initial diagnosis of primary breast cancer, pathological stage, Cell differentiation grade, staging at diagnosis based on the tumor/nodes/metastasis staging system of the Union for International Cancer Control, laboratory data during the follow-up, hormone status, parietal history, and subsequent chemo/radiotherapy were recorded. The Hb level in each month within the 1-year period following diagnosis was recorded. Patients died within 1 year or follow up less than 1 year were excluded. Stage 0 cancer was also excluded. Details of patient selection were shown in Supplementary Figure S2. The cases were followed until 12/31/2014. The primary endpoint was cancer-related death. The study was approved by the institutional review board.

### Statistical analysis

Continuous variables are reported as the mean ± standard deviation, and categorical data are reported as frequencies (percentages). Group-based trajectories with a latent class model were used to identify distinct trajectories of Hb. These models were fit using the SAS ProcTraj procedure [14, 15]. Posterior probabilities were used to assign membership to the different trajectory groups. The model fit was assessed using the Bayesian Information Criterion (BIC), and a censored normal model is appropriate for continuous outcomes [16]. The optimal number of trajectory groups was evaluated using the BIC; the number of cases in each trajectory group must exceed 3% of the total population.

Differences in clinical variables between Hb trajectories were tested using one-way analysis of variance (ANOVA) for continuous variables and chi-square tests for categorical variables. The survival curve was plotted using the Kaplan-Meier method based on the different Hb trajectory groups. A Cox-proportional hazard model was used to estimate the impact of the Hb trajectory groups on 10-year overall survival; 2-, 3-, and 5-year survival analyses were also performed to evaluate the impact of Hb trajectories at different time points. In all the analyses, a two-sided P value < 0.05 was considered statistically significant. All the statistical analyses were performed using SAS software (Version 9.4; SAS Institute, Inc., Cary, NC, USA).

## SUPPLEMENTARY FIGURES


