# Supplementary material for: Hemoglobin level trajectories in the early treatment period are related with survival outcomes in patients with breast cancer
Source: Oncotarget. 2016 Nov 29;8(1):1569–79. doi: 10.18632/oncotarget.13679 (PMC5352078; doi:10.18632/oncotarget.13679)
Supplement: Supplementary file 1 [file oncotarget-08-1569-s001.pdf]

Hemoglobin level trajectories in the early treatment period are related with survival outcomes in patients with breast cancer

SUPPLEMENTARY FIGURES

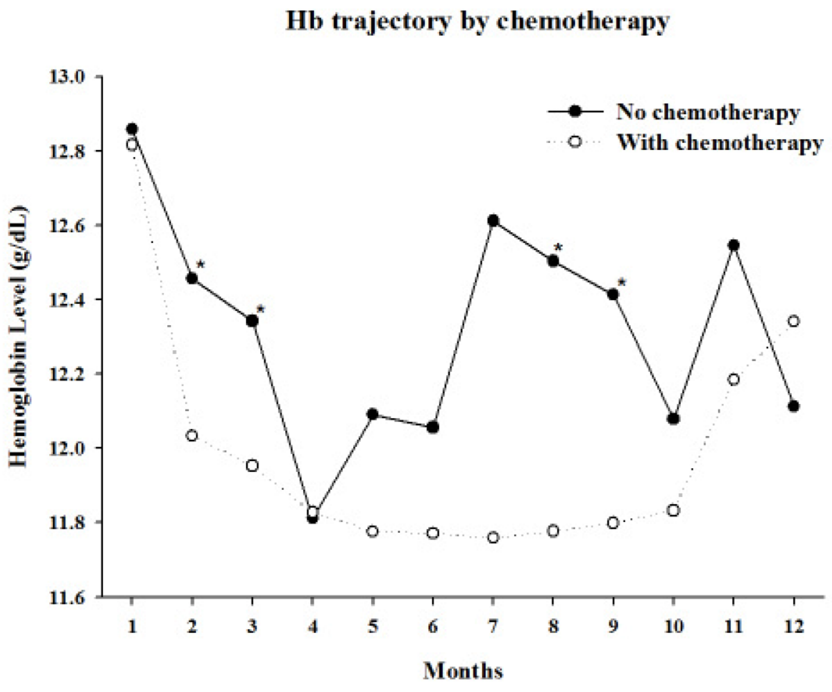

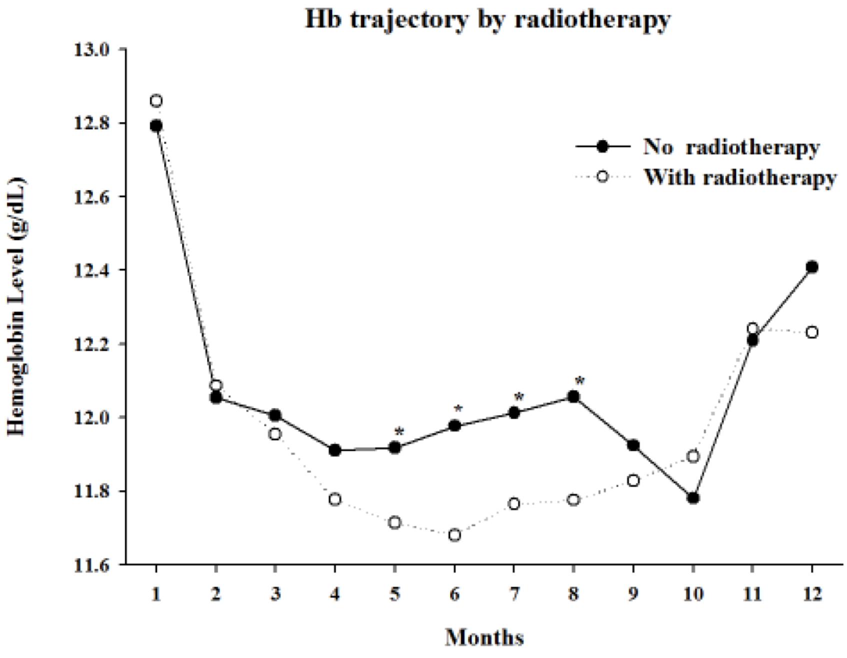

(*Continued*)

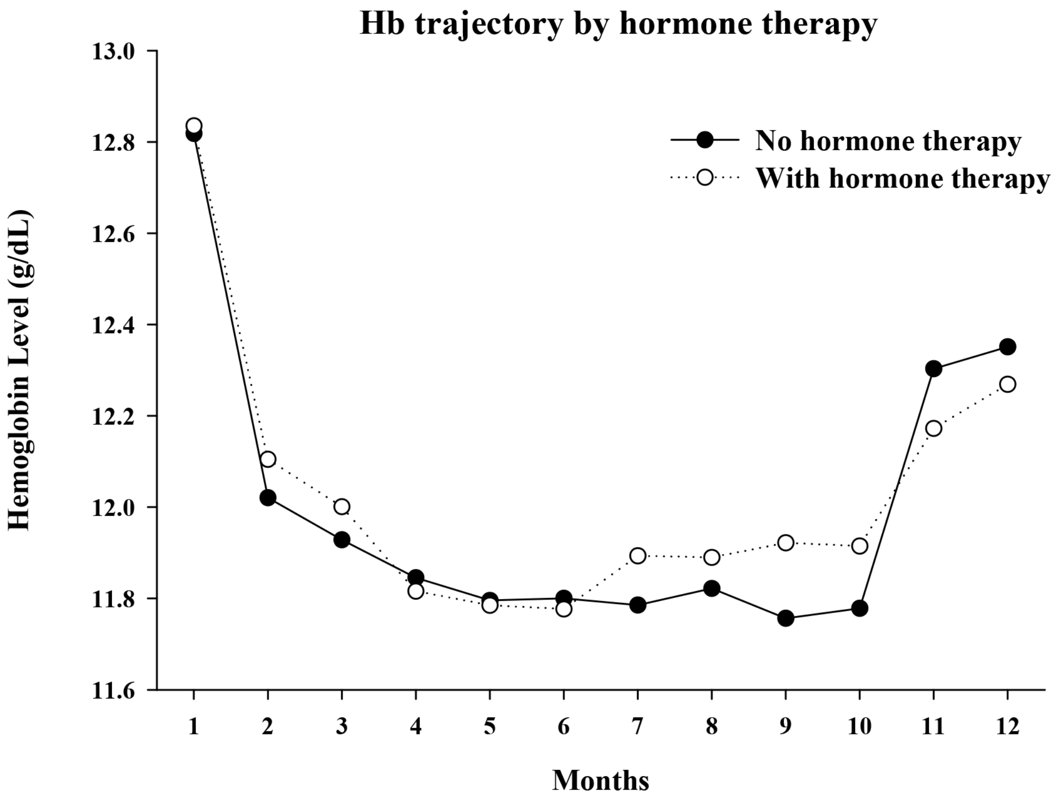

Supplementary Figure S1: Stratification analysis of treatment options vs Hb levels. Chemotherapy will significantly decrease Hb levels in month 2, 3, 8 and 9. Radiotherapy will significantly decrease Hb levels in month 5, 6, 7 and 8. The option of treatments caused temporary decrement of Hb levels, but not statistically significant in the end of the first year. Combined with table 2, we also noted that the proportion of chemotherapy and radiotherapy differed among five Hb trajectories. There was no difference of Hb trajectory in hormone therapy.

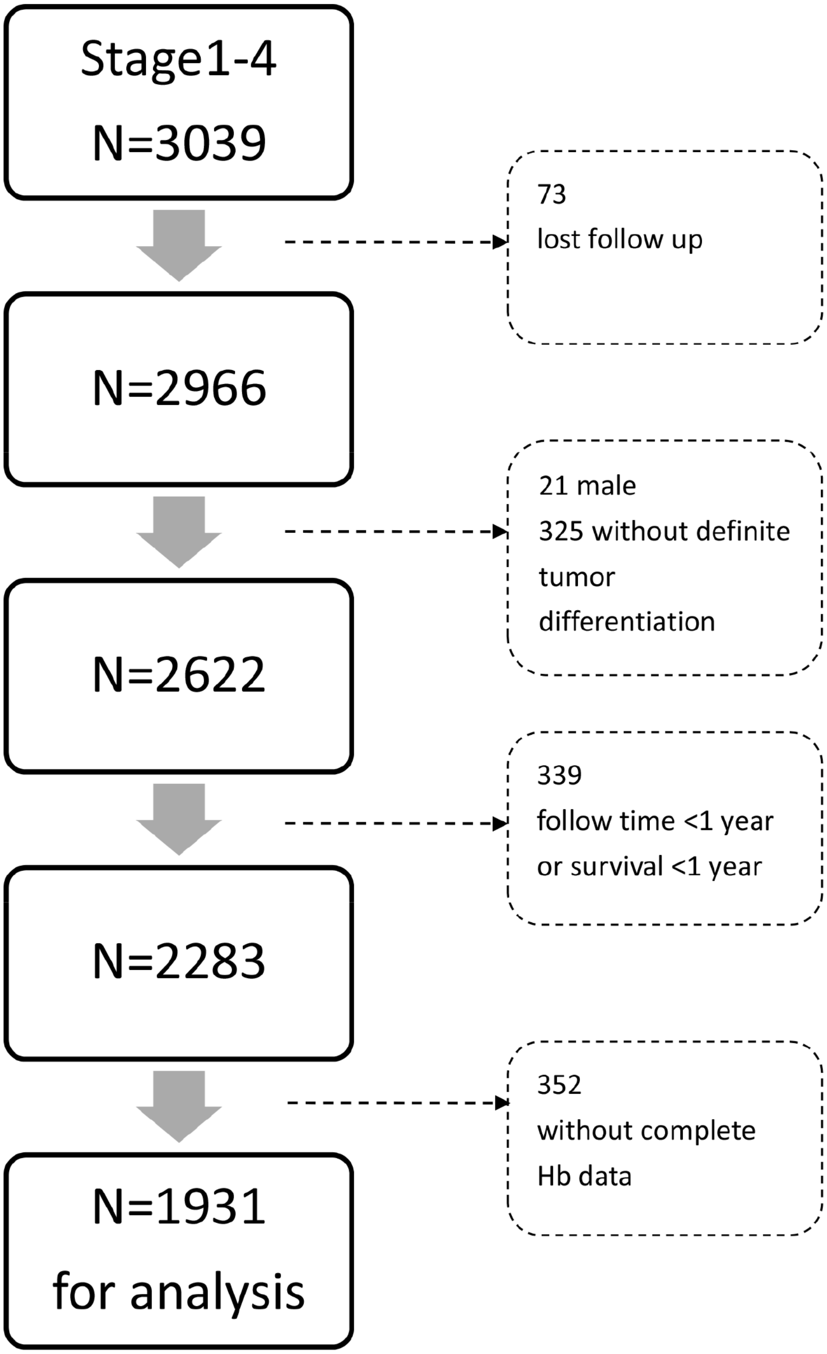

Supplementary Figure S2: Flow chart of patient selection. Overall, 3039 patients with stage 1-4 breast cancer were enrolled. Among them, 73 patients lost follow up. Patients without definite tumor differentiation (N=325) were excluded. In order to identify optimal hemoglobin trajectories, patients died within 1 year or follow up less than 1 year (N=339) were excluded. In addition, patients without complete hemoglobin data (N=352) were also excluded. Finally, 1931 patients were included for trajectory analysis of hemoglobin.
